# Diagnostic and Therapeutic Particularities of Symptomatic Melanoma Brain Metastases from Case Report to Literature Review

**DOI:** 10.3390/diagnostics14070688

**Published:** 2024-03-25

**Authors:** Adelaida Avino, Daniela-Elena Ion, Daniela-Elena Gheoca-Mutu, Abdalah Abu-Baker, Andrada-Elena Țigăran, Teodora Peligrad, Cristian-Sorin Hariga, Andra-Elena Balcangiu-Stroescu, Cristian-Radu Jecan, Adrian Tudor, Laura Răducu

**Affiliations:** 1 Discipline of Plastic Surgery, ‘Carol Davila’ University of Medicine and Pharmacy, 020021 Bucharest, Romania; adelaida.avino@gmail.com (A.A.); cristian.jecan@umfcd.ro (C.-R.J.); laura.raducu@umfcd.ro (L.R.); 2 Doctoral School, ‘Carol Davila’ University of Medicine and Pharmacy, 020021 Bucharest, Romania; abdalah.abu-baker@drd.umfcd.ro; 3 Department of Plastic and Reconstructive Surgery, ‘Prof. Dr. Agrippa Ionescu’ Clinical Emergency Hospital, 011356 Bucharest, Romania; daniela-elena.mutu@umfcd.ro (D.-E.G.-M.); andrada-elena.tigaran@rez.umfcd.ro (A.-E.Ț.); teodora.peligrad@rez.umfcd.ro (T.P.); 4 Discipline of Anatomy, ‘Carol Davila’ University of Medicine and Pharmacy, 020021 Bucharest, Romania; 5 Department of Plastic and Reconstructive Surgery, Clinical Emergency Hospital Bucharest, 014461 Bucharest, Romania; 6 Discipline of Physiology, Faculty of Dental Medicine, ‘Carol Davila’ University of Medicine and Pharmacy, 020021 Bucharest, Romania; 7 Discipline of Anatomy and Embriology, University of Medicine, Sciences and Technology “George Emil Palade”, 540139 Targu Mures, Romania; aditudo69@yahoo.com; 8 Department of General Surgery I, Targu Mures Emergency Clinical Hospital, 540136 Targu Mures, Romania

**Keywords:** cutaneous melanoma, melanoma brain metastases, metastatic melanoma, psychiatric symptoms, immunotherapy, targeted therapy, neurosurgery, SRS, multidisciplinary approach

## Abstract

The recent introduction of immunotherapy and targeted therapy has substantially enriched the therapeutic landscape of metastatic melanoma. However, cerebral metastases remain unrelenting entities with atypical metabolic and genetic profiles compared to extracranial metastases, requiring combined approaches with local ablative treatment to alleviate symptoms, prevent recurrence and restore patients’ biological and psychological resources for fighting malignancy. This paper aims to provide the latest scientific evidence about the rationale and timing of treatment, emphasizing the complementary roles of surgery, radiotherapy, and systemic therapy in eradicating brain metastases, with a special focus on the distinct response of intracranial and extracranial disease, which are regarded as separate molecular entities. To illustrate the complexity of designing individualized therapeutic schemes, we report a case of delayed BRAF-mutant diagnosis, an aggressive forearm melanoma, in a presumed psychiatric patient whose symptoms were caused by cerebral melanoma metastases. The decision to administer molecularly targeted therapy was dictated by the urgency of diminishing the tumor burden for symptom control, due to potentially life-threatening complications caused by the flourishing of extracranial disease in locations rarely reported in living patients, further proving the necessity of multidisciplinary management.

## 1. Introduction

Psychiatric conditions such as depression and anxiety are a frequent occurrence in patients diagnosed with late-stage cancer [1,2]. Conversely, surgeons treating locally advanced tumors sometimes discover that their patients have in fact long been treated for psychiatric disorders, which turn out to have been the first manifestations of metastatic brain disease stemming from a yet undiagnosed cancer.

Metastatic brain tumors represent the most prevalent intracranial masses in adults (15–25%), being 10 times more frequent than primary central nervous system (CNS) malignancies [3,4]. Their incidence has been rising with the advances in imaging techniques and the widespread availability and utilization of magnetic resonance imaging (MRI), but also because of improved peripheral disease control and prolonged overall survival (OS)due to systemic therapies. These therapies lack intracranial efficacy, and 10 to 40% of patients with solid primary tumors are expected to develop brain metastases [4,5,6].

Melanoma is the tumor third most likely to metastasize to the brain, following breast and lung cancers, and is also among the most common types of metastatic cancer diagnosed after a first encounter with a psychiatrist [7,8]. In stage IV disease, characterized by the presence of distant metastasis, the survival prospects are dismal, with the success of treatment being contingent on early diagnosis and the initiation of optimal treatment [9,10].

Melanoma accounts for approximately 1% of malignant cutaneous tumors, but it represents the most aggressive form of skin cancer, ranking in the top 5 cancers most frequently diagnosed in countries with high proportions of fair-skinned individuals [11]. Moreover, its incidence and mortality have been rising steadily for the past 60 years (an up to 3–7% increase in incidence per year, with mortality showing a milder pace), with the former being accentuated by the enhanced diagnosis of thin and in situ melanomas [12]. Numerous constitutional, clinical, demographic, and environmental factors are involved in the pathogenesis of melanoma, among which sun exposure and ambient ultraviolet radiation are responsible for 63–90% of cases [11]. Their additive yet devastating effects are reflected by the impressive density of mutations harbored by melanoma cells, which are unsurpassed by any other type of cancer presently known [12].

Melanoma diagnosis can be quite challenging in psychiatric patients and, moreover, symptom control with proper antipsychotic medication may unfortunately lead to the delayed detection of underlying brain metastases. Neuropsychiatric symptoms are more frequently associated with metastatic tumors as opposed to primary CNS malignancy, but they represent the first manifestation of a brain mass in only 18% of patients diagnosed with this pathology [3,13].

The treatment of metastatic melanoma should integrate local management and systemic therapies. Local management consists of primary tumor and metastasis resection, radiotherapy for intracranial metastases in the form of stereotactic radiosurgery (SRS), or whole-brain radiation therapy (WBR). Systemic therapies represented by targeted therapy, immunotherapy, chemotherapy and, more recently, adoptive T-cell transfer or oncolytic viral therapy, as dictated by an interdisciplinary tumor board with expertise in diagnostic and therapeutic options, are used to design an adequate treatment algorithm tailored to the patient’s comorbidities and potential side effects [10,14]. Treatment-related sequelae may augment the neuro-oncologic challenges attributed to the underlying intracranial disease, but decreasing the size of the tumor, halting its growth, or simply treating the acute mass effect can be attempted to alleviate these symptoms. The removal of brain masses amenable to local treatment is the only reliable solution for eradicating them [3,15].

Isolated oligometastatic brain disease, even when symptomatic, is extremely rare in clinical practice, since the brain is infrequently the only site of melanoma dissemination [10]. Conversely, asymptomatic oligometastatic brain disease associated with extracranial metastases represents the predominant profile of patients. Those that additionally present with neurologic symptoms have a more somber prognosis, with rapid disease progression and death from neurologic causes [10,14,16]. Most guidelines recommend prioritizing neurosurgery or SRS to address melanoma brain metastases (MBMs), with the latter applicable nowadays to lesions as small as 3–5 mm in diameter [10,14,17]. Primary and secondary tumor resection has been proven to improve outcomes for stage IV melanoma patients, with the pivotal role of cytoreduction in diminishing the immunosuppressive factors secreted by melanoma cells and therefore in restoring the immune system’s capacity for fighting malignancy being acknowledged by Morton since 1999 [18]. Nowadays, the postoperative survival rates range from 28% to 40% at 5 years if systemic targeted therapy or immunotherapy is administered [9].

We will be presenting a rare case of gigantic polypoid melanoma, an unusually aggressive variant of nodular melanoma [19]. Its discovery was delayed in a presumed psychiatric patient whose symptoms were caused by frontal melanoma oligometastases.

## 2. Case Presentation

We report the case of a 55-year-old male presenting with a painful, foul-smelling, ulcerated exophytic forearm tumor that had been growing and changing in appearance for 2 years prior to presentation; he had become increasingly isolated from society and had resigned from his profession, developing depressive and delirious disorder manifestations that had required antipsychotic medication for the previous few months. Notably, the patient disregarded the threatening nature and symptoms of this evolving mass and was referred to our plastic surgery department by a concerned relative.

**Figure 1:**
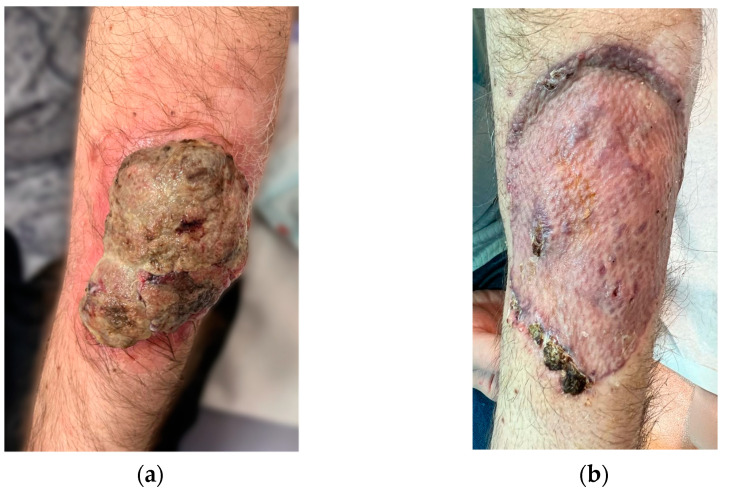
(**a**) Ulcerated, polypoid melanoma of the left forearm. (**b**) Excisional defect coverage—fully integrated graft at 1 month after surgery.

Clinical examination revealed an ulcerated, infected tumor located on the dorsal aspect of the left forearm, in the middle third. The exophytic part of the tumor, with multiple bleeding and suppurative superficial ulcerations on a grayish background, projected low over intact skin for 1 cm around the pedicle, and was extremely frail (Figure 1a).

The patient had been diagnosed with delirious disorder with mixed persecutory and grandiose delusions, and paranoid ideation making him suspicious and interpretative; this was in addition to hypomnesia, hypoprosexia, irritability and anxiety, anhedonia and apathy, hypnic anomalies, bizarre behavior, and tangential and circumstantial speech that showed no improvement after treatment with Olanzapine for 3 months.

**Figure 2:**
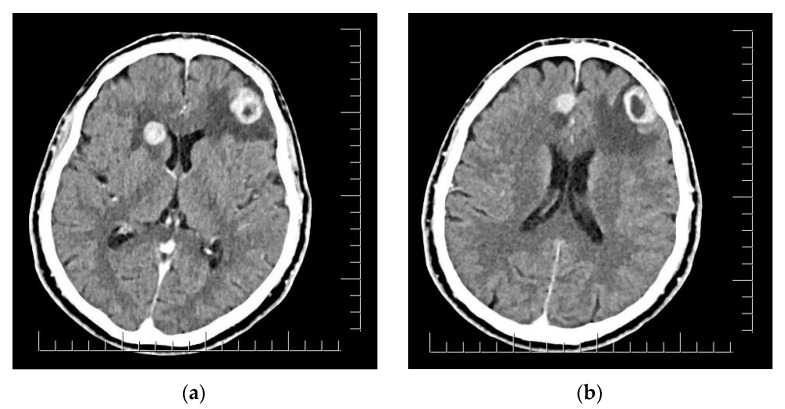
(**a**) Right fronto-insular and left frontal lobe brain lesions with adjacent vasogenic edema. (**b**) Right parasagittal and left frontal lobe brain lesions. Scale bar: 12.5 mm.

**Figure 3:**
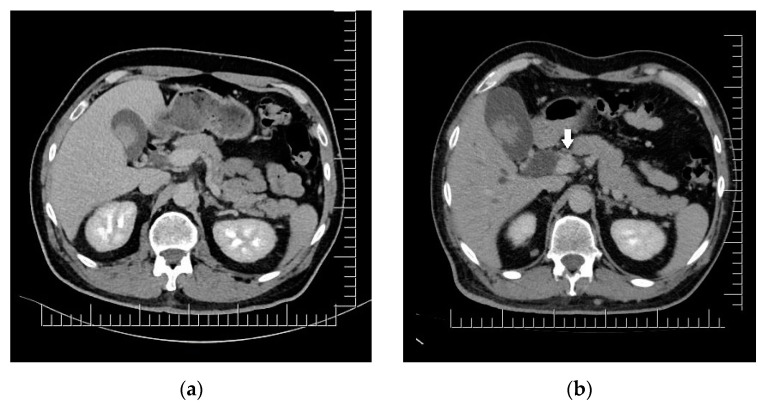
Biliary duct metastases: (**a**) Polypoid gallbladder metastasis on initial CT. (**b**) Dilation of cholecyst (containing multiple enlarged polypoid lesions) and extrahepatic bile ducts secondary to distal common bile duct obstruction though iodophilic melanoma metastasis (arrow). Scale bar: 12.5 mm.

The clinical aspect of the tumor and its association with neuropsychiatric symptoms were highly suggestive of malignancy; thus, a whole-body contrast-enhanced computed tomography (CT) examination was performed. The CT examination showed three iodophilic round lesions in the frontal lobes bilaterally and the right fronto-insular region, with adjacent finger-like vasogenic edema. The largest lesion (25.4/21.3 mm) occupied the left frontal lobe and presented a ring-like aspect suggesting a secondary substrate (Figure 2). Additional thoracic findings included lung nodules measuring 5.7 mm at most (right inferior lobe), enlarged left axillary lymph nodes (up to 24.7/15 mm), and pericardial effusion (measuring 10 mm in the superior recess and 8.6 mm in front of the right ventricle). Other findings included polypoid, dense, and slightly iodophilic lesions attached to the lateral wall of the cholecyst, measuring 29.4/16.4 mm (Figure 3a), and an oval lesion of 22.3/14.7 mm with an increased density and contrast enhancement suggestive of metastasis in the infraumbilical subcutaneous tissue.

**Figure 4:**
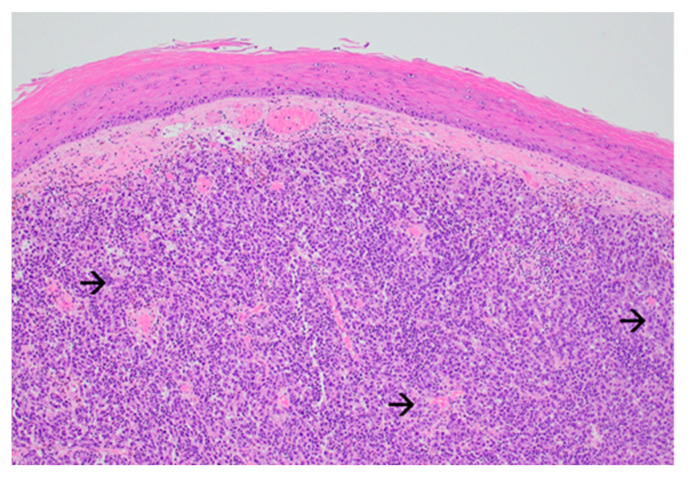
Exophytic nodular melanoma: expansive growth pattern with absence of maturation with increasing dermal depth. The tumor cells are enlarged, atypical even at this magnification, and have abundant amphophilic cytoplasm and prominent nucleoli (black arrow) (Haematoxylin and Eosin, ×40).

**Figure 5:**
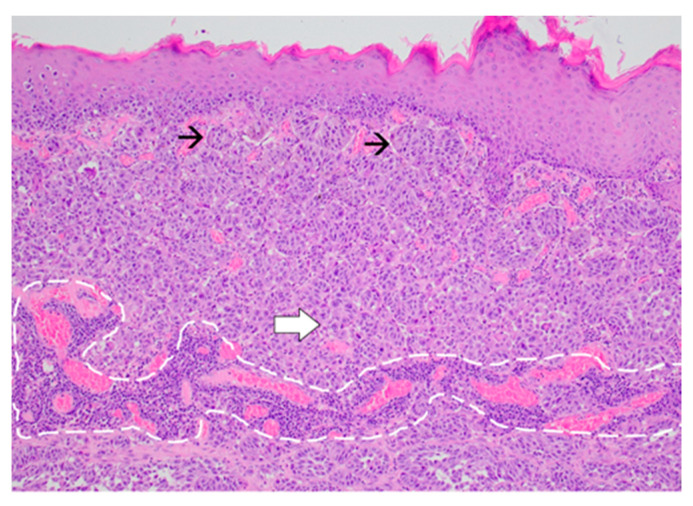
Proliferation of melanoma cells arranged in nests (black arrow) at the level of the papillary dermis and in sheets at the deeper levels (white arrow). Non-brisk tumor-infiltrating lymphocytes are noted (encircled area) (Haematoxylin and Eosin, ×20).

**Figure 6:**
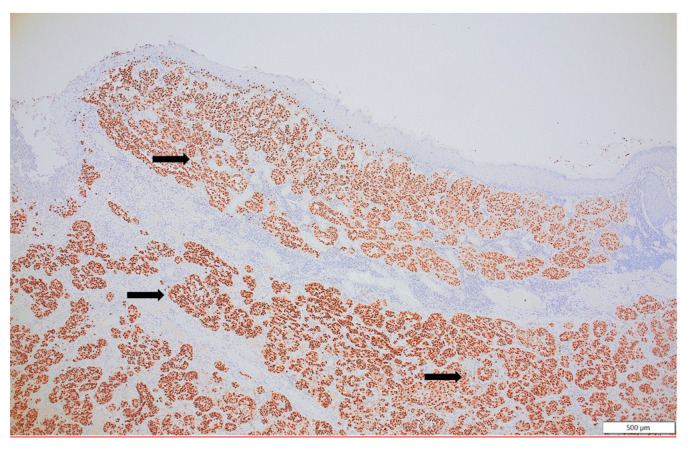
SOX 10, a sensitive and specific marker of malignant melanoma, is strongly expressed in all tumor cells (black arrow), confirming the melanocytic lineage (SOX10, ×10).

The forearm tumor was excised with oncologic safety margins of 1 cm from its apparent outer circumference, with the additional excision of two pigmented lesions located lateral to the initial defect. The dissection was carried out in the suprafascial plane since no gross invasion of the fascia underlying the tumor was observed. The defect measuring 11 cm × 8 cm was covered with a split-thickness skin graft and was secured with a tie-over bolster pressure dressing over a non-adherent contact layer of silver dressing. The postoperative course was uneventful, and the patient was discharged on postoperative day 2. Tie-over dressing rehydration with antiseptic solution and graft and donor site dressings were performed routinely on an outpatient basis. At 14 days after the surgery, 100% graft uptake and the complete epithelialization of the donor site was documented (Figure 1b).The histopathologic report established the diagnosis of invasive ulcerated nodular melanoma (Figure 4), with a Breslow index of 2.1 mm and a Clark level of V, five mitoses per square millimeter, intratumoral non-brisk lymphocytic infiltrates (Figure 5), the absence of tumor regression, and micro- or macro-satellite lesions. Analysis of a local lymph node included in the excised specimen showed the absence of malignant invasion. All the lateral excision margins proved uninvaded, with the closest being located 30 mm from the tumor; the deep excision margin was 3.5 mm away from the invasive melanoma cells. Immunohistochemistry assessment certified the presence of MELAN A, HMB45, SOX10 (Figure 6), S100, and p16 in melanoma cells and established a Ki67 index of 11%.

The tumor was staged as pT3b pN0 M1(0), R0 (stage IV cutaneous melanoma associated with low LDH levels, with complete resection of the primary tumor) according to the 8th edition of the AJCC staging guidelines [20]. Re-excision was considered unnecessary due to the larger initial excision margins. Furthermore, the BRAF gene mutation V600E was detected based on the excision specimen. The diagnosis of BRAF-positive stage IV cutaneous melanoma with cerebral, pulmonary, subcutaneous, cardiac and cholecystic metastases was established, and the case was further analyzed by the multidisciplinary tumor board of our hospital.

After the diagnosis of melanoma, the ablation of the largest symptomatic lesions (left frontal lobe and right frontal parasagittal region) through Gamma Knife SRS and microscopic neurosurgery was performed. Histopathology confirmed the metastatic nature of the lesions, but the deep, right fronto-insular lesion remained unattended. Daily Levetiracetam treatment was prescribed as a prophylaxis for post-craniotomy seizures, and systemic therapy was delayed until the remission of the postoperative cerebral vasogenic edema allowed the discontinuation of corticosteroids.

A whole-body CT scan was performed one month after brain surgery; this revealed the expansion of the subcutaneous lesion in the right paramedian infraumbilical region (measuring 21/32/33 mm) and similar looking infracentimetric subcutaneous lesions in the paramedian epigastric region and right iliac fossa. The patient returned to the plastic surgery department for the excision of these potential metastatic lesions. He presented with nausea, scleral jaundice, and increased total, conjugated and unconjugated bilirubin, indicating biliary or cystic duct obstruction. CT imaging revealed significant liver enlargement, the dilation of the intra- and extrahepatic bile ducts, and the enlargement of the cholecyst due to the presence of an iodophilic mass obstructing the distal common bile duct (Figure 3b). The patient was directed towards an interventional gastroenterology department where emergency ERCP and stent placement in the common bile duct and pancreatic duct was performed, together with bile collection and brushing; this confirmed the presence of malignant melanoma cells.

**Figure 7:**
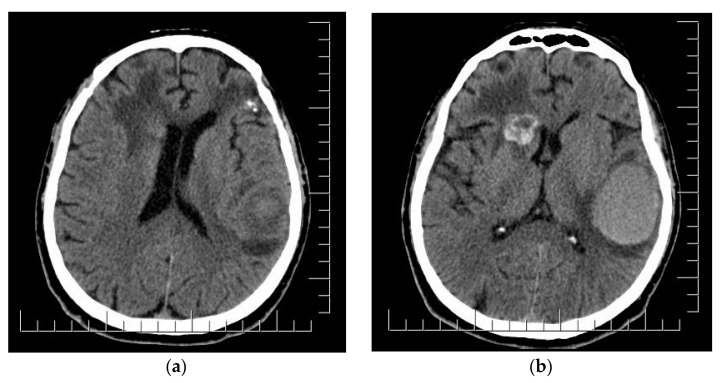
(**a**) Postoperative aspect of left frontal lobe MBM and irradiated parasagittal MBM; edema and shift of anterior midline structures. (**b**) Right fronto-insular MBM enlargement and development of cystic appearance; subacute hematoma in the left temporo-parietal region. Scale bar: 12.5 mm.

CT also showed enhancing masses in the right frontal lobe adjacent to the lateral ventricle (15/15 mm) and head of the caudate nucleus (24/21 mm), corresponding to the remaining metastasis (Figure 7). All lesions were associated with finger-like edema and a 3 mm shift in the anterior midline structures. The follow-up CT also indicated the progression of the pulmonary lesions (the lobulated nodule in the postero-basal inferior lobe of the right lung reaching 12/10 mm), cardiac involvement (two moderately iodophilic lesions occupied the right atrium and a similar sub-pericardial lesion appeared at the connection of the left inferior pulmonary vein with the left atrium), and multiple previously absent micronodular lesions suggestive of metastatic spread in the splenic subcapsular parenchyma, mediastinal and intra-abdominal fat compartments. There were also numerous nodular lesions with metastatic appearance disseminated in the subcutaneous fat in the posterior cervical, thoracic, abdominal, and pelvic region. The previously described infraumbilical mass doubled in size and became protrusive.

Although the LDH levels had previously decreased from 159 to 115 units/L after the removal of the primary tumor, increased LDH levels (265 units/L) were detected, probably simultaneously reflecting the flourishing metastatic load in the absence of systemic treatment, as well as the liver damage.

One week after the ERCP procedure, the patient began combined targeted therapy with dabrafenib and trametinib. The shrinking of all metastatic lesions, including the unoperated MBM, right inferior lobe pulmonary nodule, right atrial tumor, splenic and mesenteric nodules, and subcutaneous and muscular metastases, was observed. The patient was assessed monthly by alternating PET-CT, cerebral MRI and whole-body CT. The patient’s balance impairment and frontal lobe syndrome (amnestic and behavioral anomalies) improved 4 months after neurosurgery and the patient was able to resume his job.

Despite this spectacular recovery, after 7 months of BRAF/MEK inhibitor therapy, the patient started to experience recurrent hypoabulia, with irritability, mystic and euphoric delirious ideation, hypomnesia, circadian rhythm reversal and executive function decline, suggestive of MBM progression. Psychiatric symptoms were the first sign of recurrence under systemic therapy and were confirmed by brain imaging. With the extracranial disease under control, surgery was selected to manage the recurrent lesions because of the MBM topography and the need to avoid additive radiation damage in a patient with previous SRS. The patient was switched to immunotherapy (ipilimumab + nivolumab) after the steroid treatment was safely de-escalated. He maintained an adequate performance status in the absence of progressive disease 1 year after diagnosis.

## 3. Discussion and Review of the Literature

Polypoid melanoma is an exophytic variant of nodular melanoma, with rapid vertical growth (more than 50% of its area located above the epidermis) and limited radial spread; it was described initially by Vogler in 1958 and is sometimes referred to as pedunculated melanoma [19,21]. Showing an incidence of 2–43% in various studies, it tends to arise in younger age groups, being located predominantly on the dorsal trunk, but also in unusual locations such as the digestive tract mucosa. It is considered the most aggressive form of melanoma due to its high mitotic index and degree of cellular atypia. Its deep penetration level at the time of surgical excision causes the early invasion of the peripheral vasculature and lymphatics, leading to occult metastases and reserved prognosis [19,22,23].

The microscopic assessment of the primary tumor is essential for staging, prognosis and establishing therapeutic strategies. The Breslow index correlates with survival in stages I and II, as well as the risk of local recurrence and nodal metastasis. The Breslow index is a key factor in selecting patients for sentinel lymph node biopsy, while ulceration, the mitotic rate, lymphovascular invasion, neurotropism, and the presence of regression and tumor-infiltrating lymphocytes reflect the regional and distant metastatic potential and the immune system’s reactive capacity [12]. Biomarkers assessed through immunohistochemistry have major prognostic value as well. Among the proteins investigated in the presented case, MELAN A and HMB45 are related to the risk of recurrence; S100, p16 and SOX (the latter frequently identified in MBM) are associated with metastatic potential; and a Ki67 index over 30% is associated with a higher risk of recurrence [5,9,12].

Re-excision to achieve surgical margins that are tailored to the depth of invasion is considered curative, and it ensures the 5-year melanoma-specific survival of 95–98% in stages I and II; higher stages require additional surgery or systemic approaches to eradicate nodal or visceral disease [11,14].

Wide resection with oncologic margins of 2 cm and sentinel lymph node biopsy are necessary for polypoid melanomas when indicated (redundant for stage IV melanomas) [14]. This results in relatively large skin defects that are unsuitable for primary closure in areas with reduced skin laxity, high visibility, and significant functionality, such as the face and limbs [24]. The forearm possesses a limited amount of tissue for local rearrangement, so we opted for rapid defect coverage with a skin graft, with minimum donor site morbidity as well as simple postoperative care requirements; this allowed the early hospital discharge of the patient, with limited compliance and special psychologic requirements. Graft integration occurs within 2 weeks with classic methods such as the tie-over bolster dressing. Negative-pressure wound therapy can theoretically accelerate this process, being acclaimed for its wound-healing properties; although the presence of malignancy has generally been considered a contraindication of this technique due to its angiogenetic properties, recent research has proven its safety after complete tumor resection and its ability to decrease surgical wound complications [25].

Complex interventions such as regional, perforator or pedicled free flaps with skin paddles (preferably fasciocutaneous for suprafascial limb defect coverage) can rarely achieve dimensions comparable to those of skin grafts without the significant morbidity of the donor site; they also involve higher rates of complications (necrosis, dehiscence, infection, flap loss), thus prolonging the hospital stay. A notable exception is the keystone flap, a popular choice for large trunk and limb defects, including those spanning important joints, where grafts may produce contraction [26]. Plastic surgeons often approach primary melanoma excision through basic procedures so they can achieve stable soft tissue coverage and prompt healing while maintaining the contour, texture, color, and function of the region, preferring to preserve more complex reconstructive options for post-re-excision reconstruction [24].

It is widely acknowledged that the initial oncologic workup of malignancies with a penchant for the development of brain metastasis, such as small cell lung carcinoma (SCLC), advanced non-SCLC and advanced melanoma, should include a screening cerebral MRI [6]. Any physician dealing with psychiatric manifestations, particularly neurologists and psychiatrists, should suspect a secondary etiology of the patient’s condition, especially when it appears refractory to treatment and the manifestations recur after previous documented control. Psychiatric guidelines state that neuroimaging should be performed in the previously mentioned situations, but also in the context of new-onset psychosis, mood and memory symptoms, personality changes, the occurrence of new or atypical symptoms, and anorexia without body dysmorphic symptoms [3,27]. Especially in patients with no personal or familial psychiatric pathology before the age of 50, these psychiatric manifestations can be the first clue that a brain tumor, including metastatic cancer such as melanoma, is developing.

Brain metastases appear as single intracranial lesions in only 20–40% of patients and require careful differentiation from primary CNS neoplasms (gliomas or lymphomas), but also benign lesions (vascular malformations, abscesses and meningiomas) [6]. Their characteristic appearance is represented by well-demarcated margins, contrast enhancement, and distribution at the subcortical gray–white junction. These lesions are frequently associated with peritumoral vasogenic edema. A study by Spanberger et al. shows that reduced edema is associated with increased levels of brain infiltration and therefore decreased survival [5,28]. The cystic aspect observed on our patient’s CT, with a smooth, enhancement ring sur-rounding a spherical T1 hypointense centeris specific for growing metastases and can also predict a slower response to oncologic treatment and lower radiosensitivity, as observed by Wang et al. [6,29].

MBM arises in 12–20% of melanoma patients, although autopsy series place the proportion at 36–54% of patients with advanced melanoma [9]. They are usually associated with extensive visceral disease, with over 90% of patients having extracranial metastases as well; about a third of stage IV melanoma patients already have MBM at the time of diagnosis [10,15]. The risk factors for MBM development comprise the presence of other visceral diseases, lymph node involvement, male gender, location (head, neck, acral lentiginous and mucosal melanoma), elevated serum LDH levels, a high Breslow thickness, and nodular and ulcerated primary lesions [9,10,15,30].

The most recent AJCC staging edition established the M1d designation for CNS metastasis, with the most somber prognosis of all the M1 categories in the absence of treatment [20,31]. Despite the recent advances in understanding the biology and immunology of melanoma, which has doubled the 4-year survival rates (from 7% to 14%), the median OS of these patients rarely exceeds 2 years [15,31]. Death from melanoma is attributed to the evolution of metastases in 20 to 54% of cases, this proportion being significantly higher in the context of cerebral metastases, which directly cause death in 60–70% of patients and contribute to it in up to 95% of those with documented brain lesions [9,10,32].

CNS melanoma metastases are generally distributed across the areas receiving the highest amount of blood flow, starting with the cerebrum (frontal lobe), while the cerebellum, brainstem and spinal cord are less often affected [9]. Less than half present as solitary lesions that cause neurological symptoms corresponding to their location [10]. Metastases smaller than 1 cm tend to remain asymptomatic, but non-specific complaints or neurobehavioral changes can be encountered in the presence of tumors ranging from 1 to 4 cm in diameter [9]. The MBM detected in our patient corresponded to this category and, moreover, the psychiatric symptoms caused by the MBM were the first hallmarks of metastatic melanoma. However, he did not complain of the common symptoms associated with MBM, like headaches (classically starting in the early morning and being accompanied by nausea or visual changes), or focal neurologic defects, either isolated or in pathognomonic clusters (tinnitus, vertigo, sensorineural hearing, facial nerve deficits, diplopia, incoordination, hemiparesis or expressive aphasia defects) [3,9,33]. Melanoma metastases typically exhibit a greater propensity for hemorrhage (occurring in 33–50% of cases) and seizures (25% of cases) in contrast to metastases of other histologic types, the latter being the presenting symptom in about 15% of cases [9].

A decline in neurocognitive function (affecting up to 90% of patients with cerebral metastases) and neuropsychiatric symptoms (arising in 78% of brain tumor patients) were more obvious in our patient’s case than physical signs and symptoms [6,13]. Neurocognitive impairment can be especially striking in undiagnosed melanoma patients in contrast with other types of cancer due to the relatively younger age groups affected (melanoma is among the most common type of cancer diagnosed in young patients) [12,34]. Therefore, their neurocognitive decline is hardly attributable to more benign causes such as aging, dementia or stroke [6].

Frontal lobe tumors can cause psychiatric manifestations, ranging from mood symptoms to personality changes, that are hardly mitigated or occur despite adequate medication and therapy due to the disruption of the underlying cerebral networks by the expanding malignant process [3,27,35]. Several reviews by Madhusoodanan et al. have revealed that left frontal lobe tumors often present with akinesia and depression. Depression affects 44% of patients with brain tumors of all etiologies and is associated with functional and cognitive impairment, and a reduced quality of life and survival [3,27,36]. Right frontal lobe tumors determine maniac characteristics such as euphoria and the underestimation of negative situations, including the significance of one’s illness [3,35]. Alongside apathy and abulia, these manifestations can greatly impact the patient’s compliance with treatment and follow-up, as demonstrated by the case we presented and in consonance with the discoveries of Madhusoodanan, Benros and their collaborators [3,8,35].

Psychiatric manifestations resolve after the removal of the tumor or can be brought under control with complementary psychiatric interventions after decreasing the tumor’s size or halting its growth [3]. Aizer et al. warn that additional neurocognitive decline and behavioral symptoms can still arise as side effects of most treatments, including resection, radiation, chemotherapy, and immunotherapy, mainly due to the acute mass effect; this requires a specific treatment for increased intracranial pressure or hydrocephalus [6].

Treatment-related sequelae may augment the neuro-oncologic challenges attributed to the underlying intracranial disease. It is imperative to distinguish treatment-related sequelae from the signs of tumor recurrence [15]. This pathologic array requires diligent management through supportive medication, ranging from steroids, antiepileptic drugs, analgesics, and psychiatric control with antidepressants, antipsychotics, mood stabilizers and anxiolytics. Uninterrupted psychiatric surveillance is a vital part of the management of patients with symptomatic MBM [3].

Many researchers agree that upfront surgical resection provides a therapeutic advantage before starting immunotherapy; for example, a comparative study by Alvarez-Breckenridge et al. proved that this sequence could lead to an average survival of 22.7 months compared to immunotherapy alone, at 10.8 months, or followed by surgery, at 9.4 months [15,37,38,39]. The surgical excision of the MBM had several advantages for our patient. Firstly, the correct pathologic staging (pM1d) was achieved by confirming the metastatic identity of the cerebral masses. Secondly, as a palliative intervention, surgery improved the patient’s quality of life, prevented phenomena caused by tumor growth and increased intracranial pressure (generalized seizures, headaches, nausea, visual disturbances), and led to a reduction in the immunosuppressive factors secreted by malignant cells to ensure a more efficient antitumor response with adjuvant systemic therapy.

The latest EADO guidelines cite a few neoadjuvant trials providing evidence that patients with resectable metastases, particularly stage III melanoma, might derive numerous benefits from medical treatment prior to surgery [14]. Downsizing metastases decreases the aggressiveness and difficulty of surgery, potentially leading to a complete response, which alleviates the need for major surgery. Neoadjuvant therapy could provide reliable insights on the response of tumors to systemic medication to personalize the postoperative adjuvant treatment [14]. Thompson et al. estimate that surgery is likely to maintain a crucial role in advancing the systemic treatment of melanoma metastases, since assessing tumor histology is essential to understanding the mechanisms of response and resistance to medical therapy to develop more effective molecularly targeted therapies [40].

Neurosurgery can generally be performed for up to 3–4 brain metastases (defining oligometastatic disease, illustrated by our patient’s case) and represents the primary treatment in the case of a solitary, surgically accessible or symptomatic lesion associated with vasogenic edema or the mass effect. It is particularly effective for large, rapidly progressive tumors that are located in critical areas where SRS is more hazardous [10,15,41]; it is also the most reliable method of decompressing the damaged areas and alleviating the associated debilitating symptoms before they become permanent [10,15,40,41].

Combining two local treatment options capable of extending OS, namely neurosurgery and SRS (irradiation of the resection cavity, as proposed by Mahajan et al., or primary treatment of a limited number of additional lesions, as recommended by Akanda et al.), has also shown superior local control and lower cognitive decline compared to WBRT [6,42,43,44]. WBRT used to be the standard radiotherapy procedure applied before the development of SRS [14,15,44]. Nowadays, considering its extreme neurotoxicity, negative neurocognitive effects, decreased performance status and lack of clinical benefit in oligometastatic disease in comparison with alternative therapies, its indications have been reduced to multiple symptomatic brain metastases (more than four or those that are difficult to address through SRS or surgery), carcinomatosis or leptomeningeal disease [10,15,40,44,45,46,47,48]. Chemotherapy and WBRT are almost obsolete treatment options, and are used as a last resort for patients with an adequate performance status, unresponsive tumors or prohibitive toxicity from other treatments [14].

The standard craniotomy indications for MBM enumerated by Kreidieh et al. are as follows: diagnostic uncertainty based solely on imaging and observation (surgery provides tissue samples for diagnosis and molecular testing), symptoms unresponsive to steroids, bulky metastases (>3–4 cm), and solitary MBM in the absence of extracranial disease [15]. The presence of symptoms, the performance status, comorbidities, primary tumor characteristics and staging usually determine the decision to operate on certain patients [10,40,41,45,46,47]. Craniotomy has a mortality rate of between 1 and 3%, and can be associated with significant risks, including neurocognitive decline, leptomeningeal spread, and complications (wound infection, encephalitis, hematoma, hydrocephalus, edema, or seizures) that may require the postponement of the administration of oncologic treatment [15,40,41,48]. Nowadays, technical advances in neurosurgery, such as keyhole craniotomies and tubular retractors positioned with neuronavigational systems, limit these risks and enable surgery to be performed for multiple MBMs in areas previously accessible only through SRS [49].

SRS is an advanced, highly precise radiotherapy technique used in conjunction with high-resolution MRI; it relies on the delivery of concentrated, high doses of radiation to one or more metastatic brain lesions of otherwise radio-resistant tumors such as melanoma, with minimum damage to the normal adjacent tissue [15]. SRS can be employed as an independent primary local therapy in the form of Gamma-knife, X-knife, or Cyber-knife, allowing a swifter transition to systemic therapy compared to classic surgical resection [6,10,40,50]. SRS can be used as a salvage procedure after ineffective systemic treatment when the number of MBMs remains below 5–10 and their size is below 3 cm; in this case, the OS can be prolonged by the introduction of systemic therapy (50% and 27% survival rates at 1 and 2 years, respectively) [17,31]. Radiation necrosis (a delayed inflammatory-treatment-related imaging finding associated with brain irradiation) occurs within 3 months to 3 years after SRS and can be challenging to discriminate from tumor recurrence on CT imaging. Radiation necrosis may be associated with significant neurologic morbidity (psychomotor slowing, seizures, sensorimotor deficits, and language impairment), to the point of requiring corticosteroids/anti-VEGF antibody (bevacizumab) or neurosurgery for symptom control and histologic confirmation [6,15,31,51,52].

According to Gutzmer et al., the four main factors impacting the therapeutic sequence of MBM patients are as follows: the number of brain metastases, the presence and extent of extracerebral disease, MBM-related symptoms, and the BRAF V600 mutation status [10]. Four categories of patients are thus established, with specific treatment algorithms applicable according to the assessment of the MBM status. As the burden of metastatic disease increases, drug-resistant malignancies such as melanoma are best approached through combination therapy, with combined pathway blockade (PD-1 + CTLA-4 inhibition or BRAF + MEK inhibition), or local treatment followed by systemic treatment (which can induce a significant intracranial response even upon local recurrence) [10,14,15,37,53,54].

The presence of MBM associated with psychiatric symptoms as the incipient manifestation had a major impact on the treatment sequence of our patient. It delayed the diagnosis of the primary tumor (through apathy and abulia, social isolation, not seeking medical care). The necessity of optimizing his neuropsychiatric status before beginning systemic therapy postponed its beginning until the end of post-neurosurgery convalescence, while the extracerebral metastases advanced and required further interventions (bile duct stent placement). All these lesions and the remaining cerebral disease responded promptly to combined targeted therapy; however, in accordance with the current literature, its efficacy was limited to a few months [37]. The reported intracranial recurrence rates range from 48 to 55%. Although 20–40% are local recurrences [10], repeated treatment through microsurgery or SRS can still increase the OS, featuring better prognosis in patients with a good performance status and stationary systemic metastases [55]. Re-irradiation can be attempted if the extracranial disease is under control, but re-operation, which was also performed when recurrence was detected in our patient, may be more appropriate if radiation necrosis is suspected as a differential diagnosis to recurrence [15,56,57].

Two exceedingly rare metastatic locations, confirmed by PET-CT scans, were identified in our patient: the biliary tract and the heart. The lesions displayed significant regression during combined targeted therapy. Biliary tract metastases are detected at autopsy in about 15–20% of patients with gastrointestinal tract involvement (the 5th most common metastatic location in living patients) and in 4–20% of all stage IV melanoma patients [9]. From all cancers metastasizing to the gallbladder, melanomas are the most frequent, accounting for 50–67% of secondary gallbladder lesions, but less than 10 cases of bile duct metastatic melanoma have been documented in the literature [58,59,60]. For our patient, stent placement and systemic therapy ensured the rapid and reliable resolution of obstructive symptoms and eventually of the metastatic lesions themselves. Cholecystectomy upon diagnosis, as advised by Patel et al. for symptomatic or single, localized lesions of the cholecyst, could prevent downstream dissemination to the common bile duct [58,60].

The heart occupies the 6th place in the top sites of melanoma metastasis, with the right chambers more often affected [9]. Our patient belongs to the fewer than 2% of cases detected antemortem (autopsy series show cardiac melanoma metastases in up to 40–55% of patients), presenting bilateral atrial metastases (right intracavitary and left subpericardic); this remained asymptomatic and responded to systemic therapy [9,61].

Targeted therapy is available for melanomas exhibiting activating mutations of the BRAF gene encoding a serine/threonine protein kinase in the MAP-kinase/ERK signaling pathway. This gene is detectable through genetic testing in 45–50% of melanoma patients (V600E is the most widespread variant, with the others generally being associated with more aggressive disease) [41,62,63]. Combined BRAF and MEK inhibition guarantees an early and profound response (up to 80% size reduction) in the case of bulky, symptomatic, or rapidly progressive disease. MBMs exhibit particularly high rates of initial response owing to the high concordance between their BRAF mutational status and primary tumors compared to non-CNS metastases [31,37,54]. However, the duration of response is limited to about 10–14 months, with a median OS of 2 years; this is caused by MAPK pathway reactivation from mutations in upstream NRAS, BRAF truncation or amplification, the overexpression of genes including COT, or mutations in the downstream MEK1 kinase. Resistance develops faster in the case of symptomatic MBM [10,37,64,65,66]. Dabrafenib + trastuzumab, the combination administered to our patient, appears to be the most effective, achieving intracranial response rates between 44 and 59%, with a solid PFS of 6.5 months, as mentioned in Kreidieh’s analysis; this is superior to vemurafenib + cobimetinib or encorafenib + binimetinib [15,67,68,69]. Lower LDH levels and less than three disease sites predict favorable outcomes, while elevated baseline levels of BRAF mutant circulating tumor DNA (ctDNA) or hepatocyte growth factor (cHGF) indicate a shorter survival duration [37,70].

Immunotherapy, the second line of systemic treatment offered to our patient, constitutes the standard treatment for BRAF-wild melanomas, as well as the treatment forBRAF-mutated metastatic melanoma after the effect of targeted therapy has worn off [6,37]. It aims to induce non-specific immune stimulation through immune checkpoint-inhibiting monoclonal antibodies, which are exemplified by ipilimumab (anti-CTLA-4), nivolumab (anti-CTLA-4), relatlimab (anti-LAG-3, the newest agent) and pembrolizumab (anti-PD-1) [14]. The latter has so far obtained the most impressive clinical outcomes, with 38.8% response rates in inoperable stage IV melanoma patients and a durable 3-year intracranial response. Ipilimumab and nivolumab are the preferred combination regimen nowadays, achieving RFS at 2 years of 70% in the IMMUNED trial [14,15,53,71,72,73]. Notably, immunotherapy cannot be administered to patients suffering from autoimmune disorders or undergoing immunosuppressive therapies; this includes the corticosteroids required to alleviate neurologic symptoms or in the setting of neurosurgery, which is the reason why it was not used as a first-line therapy in our patient’s case. According to the BREAK-MB study, the intracranial response to immunotherapy appears to be influenced by the type of BRAF mutation identified, which places our patient with V600E BRAF-mutant melanoma previously treated with BRAF inhibitors in a rather favorable category, with a 31% intracranial response rate; this is opposed to V600K BRAF-mutant melanoma patients, who responded in only 22% of cases [74].

Although at present the sequential application of immunotherapy and targeted therapy is more frequent, treatment conversion occurring when progressive disease is detected or as patients enroll into clinical trials, and novel schedules to determine the appropriate time to switch are being actively investigated [37,75]. Concomitant immunotherapy with targeted therapy is feasible in BRAF-mutated melanoma patients, being able to overcome each other’s pitfalls while contributing to a more favorable tumor microenvironment at the cost of additive side effects (severe hepatic toxicity and gastrointestinal events) [37]. Triple therapies are showing promising results in the TRICOTEL study (atezolizumab + cobimetinib + vemurafenib) and TRIDeNT trial (nivolumab + dabrafenib + trametinib), as well as dabrafenib + trametinib combined with novel anti-PD-L1 agents such as pembrolizumab, spartalizumab or durvalumab.Some of these triplets allow a safe reduction in the corticosteroid dose in MBM patients and therefore potentiate the benefits of immunotherapy [76,77,78,79,80,81,82].

Several retrospective analyses by Ascierto et al. have highlighted that only patients managing to complete 6 months of targeted therapy without progression (the presented case being close to this threshold) achieve significantly higher response rates with subsequent immunotherapy compared to those with rapid relapse under treatment. Previous targeted therapy may decrease the activity of checkpoint inhibitors through the selection of more aggressive melanomas and possible tumor biology alterations associated with the development of resistance to BRAF/MEK blockade [37]. Most data indicate that, unless rapid response is imperative to control extensive or symptomatic metastatic disease, immunotherapy followed by targeted therapy is more suitable for BRAF mutant melanoma patients, providing 3.7–5.2 years of additional survival compared to sequences starting with BRAF/MEK inhibition [83,84].

Novel therapeutic targets are being proposed as the distribution of cellular populations in MBM is unraveled and single-cell RNA, or whole-exome sequencing, succeeds in highlighting the metabolic states associated with an enhanced response to immunotherapy. On the contrary, immune evasion and the increased risk of brain metastases are linked with the loss of the PTEN gene, the expression of PLEKHA5, CCR4 and Angptl4, the activation of the oxidative phosphorylation and PI3K-AKT pathways, and the differential expression of TIMP-1,2,3 [31,85,86,87,88].

Further deciphering the cellular landscape of MBMs may help to determine more dysfunctional cells and pathways that can be turned into therapeutic targets in order to hinder the development of cerebral metastases. Future clinical trials may also be designed to account for the presence of these variations and subsequent response nuances. Molecular markers are starting to be considered as prognostic factors in decisional algorithms guided by scores such as the Melanoma mol-GPA. In total, 53 proteins associated with melanoma metastasis and recurrence have been identified; as such, the era in which molecular assessments will become an established practice supported by guidelines is already in sight [89,90,91].

Finally, considering the complexity of metastatic melanoma management, as illustrated by the current literature and considering the case we reported, we wish to draw attention to early diagnosis by emphasizing the vital role of basic physical examination in detecting melanoma by applying the ABCDE criteria for skin tumors. This potentially life-saving practice should be part of any medical specialist’s evaluation of their patient’s condition, especially when facing patients with limited contact with the healthcare environment.

## 4. Conclusions

Diagnosing cutaneous melanoma can be exceedingly challenging in certain categories of patients, including those with psychiatric disorders, which exceptionally represent the first hallmark of metastatic cancer. Comprehensive screening for metastasis is an essential step after the histopathologic confirmation of melanoma, and further management should be pursued in accordance with international guidelines, as well as the recommendations of an interdisciplinary tumor board. Psychiatrists play a crucial role in identifying secondary brain tumors, which are the main cause of psychiatric disorders in patients who are otherwise reluctant to seek professional medical advice.

## Data Availability

The data presented in this study are available on request from the corresponding author due to privacy and ethical restrictions.
